# Bennet on Uterine Pathology—Gardner on Sterility

**Published:** 1856-11

**Authors:** 


### BOOK NOTICES.

*Bennet on Uterine Pathology. Gardner on Sterility.*

We are indebted to Blanchard & Lea, of Philadelphia, for the first of the above works; and to Dewitt & Davenport, of New York, for the second.

The little work of Dr. Bennet is devoted mainly to those questions in regard to which there is a diversity of opinion among uterine pathologists, and designed especially to maintain his own doctrines, as enunciated in his previously published work. Those interested in these *questions vexatæ*, will read these seventy-five pages of Dr. Bennet with pleasure.

The first chapter of Dr. Gardner's work is devoted to the Physiology of Generation; the second to the Pathology of Sterility, and the third to the Therapeutics of Sterility.

Among the causes of sterility, the author enumerates failure from any cause of the semen to enter the vagina or uterus. The destruction of its vitality by acrid, vitiated syphilitic, blenorrhagic or cancerous discharges, or its removal by immense leucorrhœal exhalations.

The author also attributes sterility, in a large number of cases, to disease of the os cervix, or cavity of the uterus, and, finally, to disease of the fallopian tubes or ovaries.

We have not time to follow our author in his examination of these questions; some of them have been the subjects of much controversy among pathologists.

The chapter on the Therapeutics of Sterility, contains nothing essentially novel or new, and we will not weary our readers by a repetition of what they will find in most of our standard works.

Both of the above works are for sale by Keen & Lee.

